# Permanent Strain Engineering of Molybdenum Disulfide Using Laser-Driven Stressors for Energy-Efficient Resistive Switching Memory Devices

**DOI:** 10.3390/nano14231872

**Published:** 2024-11-22

**Authors:** Heeyoon Jang, Seok-Ki Hyeong, Byeongjin Park, Tae-Wook Kim, Sukang Bae, Sung Kyu Jang, Yonghun Kim, Seoung-Ki Lee

**Affiliations:** 1 School of Material Science and Engineering, Pusan National University, Busan 46241, Republic of Korea; hyhy1107@pusan.ac.kr; 2 Functional Composite Materials Research Center, Institute of Advanced Composite Materials, Korea Institute of Science and Technology (KIST), 92 Chudong-ro, Bongdong-eup, Wanju-gun 55324, Republic of Korea; 3 Energy and Environment Materials Research Division, Korea Institute of Materials Science (KIMS), 797 Changwondaero, Sungsan-gu, Changwon 51508, Republic of Korea; 4 Department of Flexible and Printable Electronics, Jeonbuk National University, Jeonju-si 54896, Republic of Korea; 5 Department of JBNU-KIST Industry-Academia Convergence Research, Jeonbuk National University, Jeonju-si 54896, Republic of Korea; 6 Electronic Convergence Material and Device Research Center, Korea Electronics Technology Institute, Seongnam 13509, Republic of Korea

**Keywords:** molybdenum disulfide, strain engineering, stressor, ReRAM, low power operation

## Abstract

Strain engineering provides an attractive approach to enhance device performance by modulating the intrinsic electrical properties of materials. This is especially applicable to 2D materials, which exhibit high sensitivity to mechanical stress. However, conventional methods, such as using polymer substrates, to apply strain have limitations in that the strain is temporary and global. Here, we introduce a novel approach to induce permanent localized strain by fabricating a stressor on SiO_2_/Si substrates using fiber laser irradiation, thereby enabling precise control of the surface topography. MoS_2_ is transferred onto this stressor, which results in the application of ~0.8% tensile strain. To assess the impact of the internal strain on the operation of ReRAM devices, the flat-MoS_2_-based and the strained-MoS_2_-based devices are compared. Both devices demonstrate forming-free, bipolar, and non-volatile switching characteristics. The strained devices exhibit a 30% reduction in the operating voltage, which can be attributed to bandgap narrowing and enhanced carrier mobility. Furthermore, the strained devices exhibit nearly a two-fold improvement in endurance, presumably because of the enhanced stability from lattice release effect. These results emphasize the potential of strain engineering for advancing the performance and durability of next-generation memory devices.

## 1. Introduction

With the increasing demand for more efficient and versatile semiconductor technologies, strain engineering has emerged as a crucial method for enhancing device performance. In this technique, controlled mechanical stress is applied to modify the physical and electrical properties of materials, which enables precise control over the electronic behavior and addresses the limitations of traditional semiconductors. So far, its effectiveness has been particularly evident in silicon-based semiconductors, where it has contributed to significant improvements in both functionality and performance [1,2]. T. Ghani et al. demonstrated significant performance enhancements in the CMOS technology at the 90 nm node by applying a uniaxial strain in the transistor channels [3]. In PMOS, the epitaxial growth of SiGe in the source/drain regions induced compressive strain, whereas in NMOS, tensile strain was applied through a nitride-capping layer. These optimized strain techniques have the potential to effectively enhance the channel mobility and drive current, which is crucial for achieving power efficiency and high switching speed in advanced transistors. Recently, two-dimensional (2D) materials have gained considerable attention as promising alternatives to traditional Si for semiconductor applications, primarily because of their exceptional electrical and mechanical properties at the atomic scale, particularly in three-dimensional (3D) device architectures [4]. Unlike bulk materials, the unique structural properties of atomically thin 2D materials like MoS_2_ facilitate significant strain-tuning capabilities, which can potentially unlock new functional properties that are otherwise unattainable [5,6]. With their lattice structures confined to the atomic plane, 2D materials are highly responsive to slight deformations, allowing sub-1% tensile strain to significantly modify their band structures and electrical properties, enhancing current levels and photoresponsivity [7]. Li et al. reported that applying uniaxial tensile strain through PVA (Poly (vinyl alcohol)) encapsulation causes a 125 meV/% reduction in the bandgap, which suggests its potential for improving device performance [8]. In addition, Datye et al. reported a two-fold increase in the mobility of monolayer MoS_2_ transistors under a tensile strain of up to 0.7%, which was achieved through bending devices on flexible PEN (polyethylene naphthalate) substrates [9]. However, despite the promising properties of strain-engineered 2D materials, realizing a permanent strain in these materials remains a key challenge. Although previous research has shown that bending or stretching can induce temporary property changes in materials, the transient nature of these effects presents a major challenge for applications requiring long-term stability in strain-engineered devices. Accordingly, a range of methodologies is currently being explored to facilitate the permanent application of strain, including approaches that leverage the thermal expansion coefficient (TCE) mismatch between substrates and 2D materials [10,11,12,13,14,15]. Furthermore, Christian Martella et al. demonstrated that introducing one-directional anisotropy in MoS_2_ nanosheets via CVD on rippled SiO_2_/Si substrates enables controlled tuning of optoelectronic properties at both macro- and nanoscale levels [13]. Similarly, Hong Li et al. applied a spatially modulated biaxial tensile strain of approximately 0.5% in monolayer MoS_2_ using a nanocone patterned substrate [16].

In this study, we propose a novel strain engineering approach that facilitates permanent stress application to the desired MoS_2_ region using a stressor substrate, which induces a wrinkled structure. The SiO_2_/Si stressor substrate was precisely engineered using a laser-based selective photothermal reaction to control its surface morphology, enabling the MoS_2_ layer on top to experience a permanent tensile stress of up to 0.8% owing to the topography of the substrate. This innovative process creates MoS_2_ layers with built-in mechanical stress that provides a stable platform for exploring new physical and electrical properties that endure over time. Furthermore, we explore the application of this permanently stressed MoS_2_ as an active layer of ReRAM, the fundamental unit of neuromorphic devices. By integrating the strained MoS_2_ into these devices, the influence of permanent tensile stress on device performance was demonstrated. The results show that the strain-induced MoS_2_ active layer, where inter-element interactions are weakened by tensile stress, significantly reduced the energy required for filament generation and separation under an applied bias, which lowered the operating voltage by nearly 30%. Furthermore, the stable control of the conductive filaments resulted in marked improvements in the long-term stability of the memory devices. This approach overcomes the challenges associated with temporary strain and also offers valuable insights into how strain-engineered 2D materials can be leveraged for practical scalable device applications.

## 2. Materials and Methods

### 2.1. Materials

Ammonium tetrathiomolybdate ((NH_4_)_2_MoS_4_, 99.97% trace metals basis), N,N-Dimethylformadmide (DMF, anhydrous, 99.8%), ethanolamine (≥99.5%), and Hydrofluoric acid (ACS reagent, 48%) were purchased from Sigma-Aldrich (St. Louis, MO, USA) and used without further purification.

### 2.2. Synthesis of Graphene

The copper foil was placed inside a quartz tube within the chemical vapor deposition (CVD) system and heated to 1000 °C under an 8 sccm flow of H_2_ at a pressure of 90 mtorr. Upon reaching 1000 °C, the copper foil underwent a 30 min annealing process, maintaining the same flow rate and pressure throughout [17]. Following annealing, a gas mixture of CH_4_ and H_2_ was introduced at flow rates of 24 sccm and 8 sccm, respectively, at a pressure of 460 mtorr for 30 min. The sample was subsequently cooled to room temperature at a rate of 10 °C/s, with H_2_ flowing at 90 mtorr, resulting in graphene growth on both sides of the copper foil.

### 2.3. Synthesis of MoS_2_

A 0.03 M precursor solution was prepared by dissolving 0.05 g of (NH_4_)_2_MoS_4_ in a solvent mixture of DMF and ethanolamine in a 9:1 volume ratio, with a total volume of 0.54 mL. The solution was spin-coated onto a cleaned SiO_2_/Si wafer using a two-step process: 500 rpm for 10 s followed by 3000 rpm for 30 s. After spin-coating, the substrate was baked at 150 °C for 3 min to remove residual solvents. Thin films were synthesized through CVD. The substrate was annealed at 500 °C for 30 min in a H_2_ gas atmosphere, followed by crystallization at 800 °C for 30 min in a H_2_S gas atmosphere.

### 2.4. Fabrication of the Stressor

A pulsed fiber laser (λ = 1.06 µm) was employed to irradiate a p+ Si wafer covered with a 300 nm SiO_2_ layer. The laser parameters were calibrated as follows: focal length, 7.9 cm; scan speed, 500 mm/s; frequency, 20 kHz; and power, 5.4 W. The surface morphology was controlled by adjusting the laser-scribing width.

### 2.5. Fabrication of ReRAM

To fabricate the electrode pad, a 5 nm Cr adhesion layer and 50 nm Au layer were thermally evaporated onto the stressor. Photolithography was then employed to define the electrode pad, followed by etching of Au and Cr layers. The 1 nm graphene bottom electrode and 3 nm MoS_2_ resistive switching layer were subsequently transferred onto the line-patterned stressor. First, to transfer graphene onto the stressor substrate, Polymethyl methacrylate (PMMA) solution was spin-coated onto the front-side graphene (2000 rpm, 60 s) and baked at 100 °C. The back-side graphene was removed through reactive ion etching (RIE) with parameters of 100 W, 20 sccm O_2_, for 5 s. The copper was then etched away in an ammonium persulfate solution ((NH_4_)_2_S_2_O_8_, 4 g in 200 mL DI water). Then, the residual etchant was rinsed off with DI water. During the transfer onto the stressor, a mixture of DI water and alcohol in a 5:1 ratio was used to minimize wrinkles. Following the transfer, an annealing process was performed at 150 °C for 1 h in an inert atmosphere (~10^5^ Torr) to improve the conformity of graphene with the underlying SiO_2_ surface [18,19,20]. This process also helps to remove any residual solvents or contaminations, further improving the quality of the graphene layer and ensuring better conformity during subsequent MoS_2_ transfer. Afterwards, a PMMA solution was spin-coated on MoS_2_, and the sacrificial SiO_2_ layer was etched with HF. The MoS_2_ film was then rinsed with DI water to remove any HF residue. During transfer onto graphene, a mixture of DI water and alcohol was used. After the transfer of MoS_2_ on graphene, an annealing process was conducted, using the same procedure as the graphene transfer, to enhance the conformity of the MoS_2_ film with underlying graphene, thereby ensuring improved conformity for subsequent device fabrication. Then, graphene and MoS_2_ were etched using the reactive ion etching (RIE) process with a power of 100 W and 50 sccm of Ar flow rate for 20 s. Finally, a 50 nm Al top electrode was deposited by thermal evaporation with a pattern defined via a lift-off process.

### 2.6. DFT Calculations

First-principles calculations were performed using generalized gradient approximation (GGA) for the exchange-correlation functional within the Perdew-Burke-Ernzerhof (PBE) scheme implemented in the Quantum Espresso code. A cutoff energy of 50 Ry was applied for the plane-wave basis set, and Grimme DFT-D3 was used to account for the van der Waals interactions. For the few-layered MoS_2_ structure, a vacuum spacing of 15 Å along the z-axis was applied to eliminate the periodic boundary condition (PBC) interactions. The lattice constant for unstrained MoS_2_ was set to 3.165 Å, calculated based on the bulk MoS_2_ structure. For structural optimization, a 9 × 9 × 1 k-point grid was used to effectively sample the Brillouin zone.

### 2.7. Measurement and Analysis

The electrical properties of the synaptic device were measured using a Keithley 4200A-SCS parameter analyzer (Keithley Instruments, Cleveland, OH, USA), and current–voltage (I-V) measurements were performed on the Al/AlO_x_/MoS_2_/graphene device. The Au electrode pad connected to the graphene bottom electrode was grounded, and a DC bias was applied to the Al top electrode. The compliance current was set to 100 µA to prevent irreversible breakdown of the device. For the flat device, a voltage sweep from −4 V to +4 V (0→+4→0→−4→0 V) was applied, and for the strained device, the sweep ranged from −2.5 V to +2.5 V (0→+2.5→0→−2.5→0 V). Retention measurements were performed by applying a read voltage of 0.2 V, and the current was recorded every 10^2^ s.

## 3. Results and Discussion

**Figure 1:**
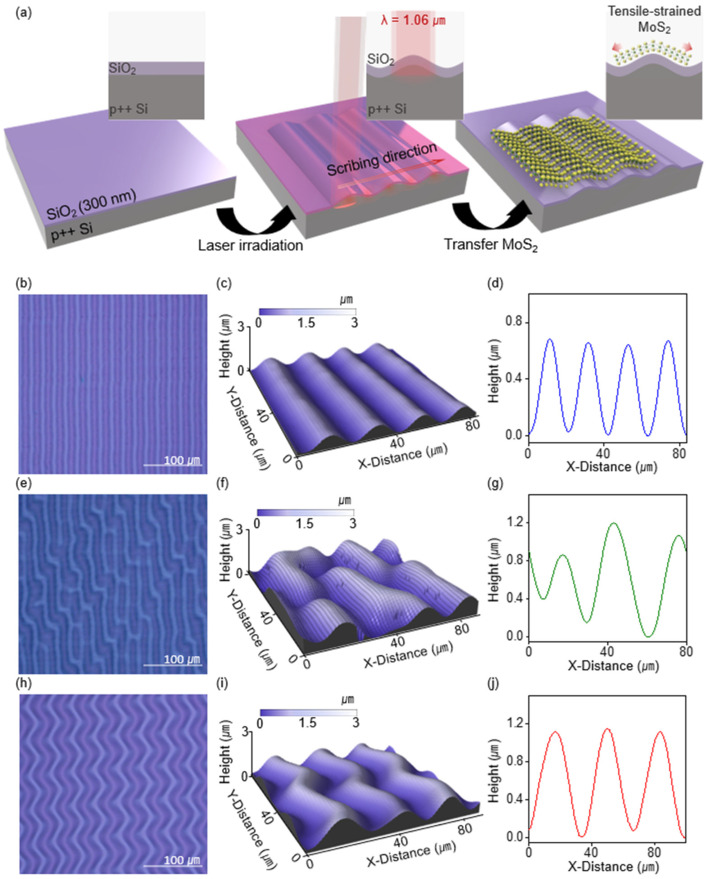
(**a**) Schematic illustration of the fabrication process for applying strain to MoS_2_ by fabricating a SiO_2_/Si stressor and using 1.06 μm laser. Optical Microscopy image of stressor with different patterns: (**b**) line, (**e**) random, (**h**) wavy patterns. Schematic of the surface morphology obtained from 3D profiler: (**c**) line, (**f**) random, (**i**) wavy patterns. Representation of the periodicity and height based on the surface morphology data: (**d**) line, (**g**) random, (**j**) wavy patterns.

Figure 1a shows a schematic diagram of the process for implementing a MoS_2_ layer with permanent strain on a SiO_2_/Si stressor substrate. The SiO_2_/Si substrate, serving as a stressor, comprises a 300 nm SiO_2_ layer fabricated using a thermal oxidation process on a Si wafer. Consequently, compressive stress is inherently present at the growth interface owing to the mismatch in the molar volume with Si and the difference in the thermal expansion coefficients [21,22]. When the stress-impregnated SiO_2_/Si substrate is irradiated using a fiber laser (λ = 1.06 μm), most of the energy is absorbed by the Si layer because of the difference in the optical absorption coefficients (α) at this wavelength [23]. The absorption coefficient of Si was approximately 1.2 × 10^3^ m^−1^, whereas that of SiO_2_ was less than 1 m^−1^. The Si layer experienced partial melting after surpassing the critical threshold, which caused the SiO_2_ layer to delaminate from the Si substrate and form a hill-like structure to relieve its internal compressive stress. During the subsequent recrystallization and expansion of Si through repeated laser irradiation, a hill-like structure was formed owing to the viscoelastic deformation of the SiO_2_ layer [24,25]. Moreover, the surface topography can be artificially manipulated into a regular pattern by controlling the periodic energy deposition characteristics of the pulsed laser. The key variables for controlling the stressor geometry include scribing width and laser power. The scribing width adjusts the overlap of the laser spots, thereby defining the regions of heat accumulation, while the laser power regulates the energy needed to melt the Si layer. Additional factors include the thickness of the SiO_2_ layer, which influences its stiffness and capacity to relieve internal stress, and the laser spot size, which further controls the overlap area of the laser [23]. This ability to control the surface morphology makes SiO_2_/Si substrates promising stressors for strain-engineering applications. When 2D materials such as MoS_2_ are transferred onto this 3D structured stressor, their mechanical flexibility enables them to absorb the substrate deformation and induce localized strain [26]. Because of its low bending stiffness and van der Waals interactions, MoS_2_ can easily conform to the surface morphology of a stressor, which facilitates stress transfer and deformation.

Figure 1b–j shows the surface morphologies of the stressor in three distinct patterns—line (b–d), random (e–g), and wavy (h–j)—which were formed by controlling the scribing width and laser power. In this experiment, the topographic pattern of the stressor was determined by the interaction between the laser power and scribing width, with the SiO_2_ thickness fixed at 300 nm and the laser spot size fixed at 20 μm. Figure S1a summarizes the outcomes under specific synthesis conditions, with colors indicating the trend of patterns. For example, when the laser power variable was fixed to 5.4 W, wavy, line, and random patterns were formed for the scribing widths 1~2 μm, 3~5 μm, and 6 μm, respectively. As the scribing width decreases, the overlap area increases, causing the melting regions to merge and transition from line to wavy patterns with progressively larger periodicity. The periodicity and height of the line and wavy patterns were 20.49 ± 0.76 μm and 0.74 ± 0.03 μm, and 42.89 ± 1.99 μm and 1.76 ± 0.08 μm, respectively (Figure S1d). The strain at the hill of each pattern was calculated using Equation (1), where *y* represents the distance from the neutral axis and ρ is the radius of curvature. Accordingly, the resulting strain of line pattern was 0.84 ± 0.07%.

(1) $$\varepsilon =\frac{y}{\rho }$$

Therefore, the process of transferring the MoS_2_ film onto the stressor was designed to apply tensile strain to a localized region as desired. The strain distribution within the line pattern stressor varies slightly depending on the location within the structure (Figure S2). To capture the significant strain effects, we focused our strain analysis specifically at the peak of the hill structures, where the strain was most pronounced. Additionally, atomic force microscope (AFM) measurements of the surface root mean square (RMS) roughness of the resulting stressor after laser treatment showed a value of 0.3 nm, which is nearly identical to the substrate value of 0.2 nm before laser treatment (Figure S3). This suggests that the laser treatment had a negligible effect on the surface roughness, indicating that it is suitable for the transfer of 2D materials.

**Figure 2:**
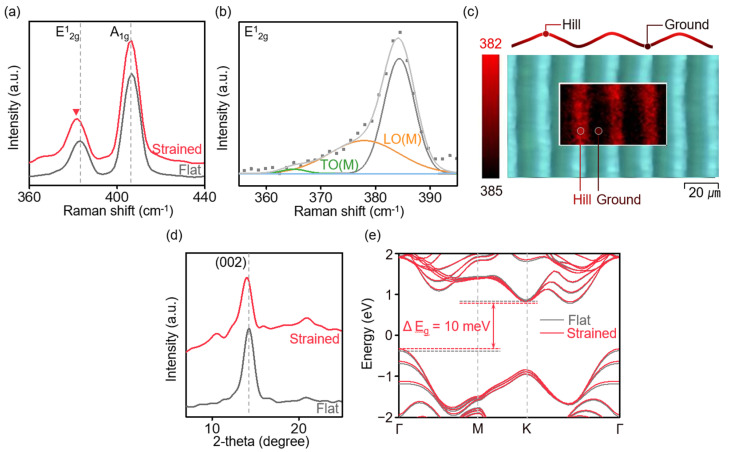
(**a**) Raman spectroscopy of flat MoS_2_ (gray) and 0.8% tensile-strained MoS_2_ (red). (**b**) Magnified $E_{2g}^{1}$ peak of strained MoS_2_ showing the split between the LO and TO mode. (**c**) Raman mapping image (50 × 30 μm^2^) of strained MoS_2_ on line-patterned stressor. (**d**) GIXRD patterns of flat MoS_2_ (gray) and strained MoS_2_ (red) at the (002) peak. (**e**) Band structure of flat MoS_2_ (gray) and strained MoS_2_ (red) obtained from DFT calculations.

Figure 2 presents the analysis of the property changes in MoS_2_ induced by the internal strain on the stressor. When a strain was applied to MoS_2_, the bonding strength and angles between the atoms changed, which influenced the vibrational modes. These modifications were analyzed by Raman spectroscopy (Figure 2a) [5,15]. For comparison, a control group, non-strained MoS_2_ (referred to as flat MoS_2_), was formed on a flat substrate. Flat MoS_2_ exhibited two dominant peaks at 383.8 cm^−1^ ($E_{2g}^{1}$) and 406.6 cm^−1^ (A_1g_), which are characteristic of tri-layered MoS_2_ [27]. In contrast, the strained MoS_2_ exhibited a red shift in the $E_{2g}^{1}$ peak to 382.0 ± 0.04 cm^−1^, indicating a shift of 1.8 cm^−1^ which reflects a softening of the vibrational mode under tensile strain. The A_1g_ peak, however, remained nearly unchanged at 406.6 ± 0.01 cm^−1^, showing no significant shift. The $E_{2g}^{1}$ peak corresponds to a vibrational mode in which the atoms oscillate in opposite phases within the basal plane. Under tensile stress, the bond lengths between the atoms increased, which reduced the vibrational energy and caused a red shift [28]. The tensile strain was estimated using Equation (2), where *γ* represents the Gruneisen parameter, which is specific to the material (for MoS_2_ *γ* ($E_{2g}^{1}$) = 0.21), and *ω*_0_ and *ω* are the initial and shifted wave numbers, respectively [29].

(2) $$\varepsilon =\frac{\omega_{0}-\omega }{2\gamma \omega_{0}}$$

The calculated tensile strain of MoS_2_ was approximately ~0.8%, which is consistent with the strain of the surface topography on the substrate. This indicates that MoS_2_ was conformed with the stressor substrate without a slip or crack. Furthermore, analyzing the $E_{2g}^{1}$ peak in the strained MoS_2_ revealed a full width at half maximum (FHWM) increase of 1.04, which indicates peak broadening (Figure 2b). For flat MoS_2_, the longitudinal optical (LO) and transverse optical (TO) phonon modes exhibited identical energies, which resulted in a single sharp peak. In contrast, in strained MoS_2_, the softening $E_{2g}^{1}$ peak can be explained through the phonon band structure [30,31,32,33]. The red shift from initial frequencies of LO and TO modes leads to symmetry breaking at the M point of the Brillouin zone and results in LO-TO splitting. This phenomenon was attributed to uniaxial tensile strain, as reported in previous studies [34,35]. Figure 2c presents the Raman mapping results of a strained MoS_2_ region (50 × 30 μm^2^). The intensity of the $E_{2g}^{1}$ peak at 382 cm^−1^ was notably stronger in the hill regions, whereas the intensity at 384 cm^−1^ was more pronounced in the ground regions, where the peak position aligns with that of flat MoS_2_. This observation suggests the absence of effective strain, including compressive strain in the ground regions, while only hill regions exhibited effective tensile strain. This distinction clearly demarcates the strained and unstrained regions within the MoS_2_ film. Figure 2d shows the grazing incidence X-ray diffraction (GIXRD) analysis, where the strained MoS_2_ exhibited a 0.2° low-angle shift in the (002) plane (c-axis) along with an increase of 0.08 in the FWHM. It was determined that the strain within crystal increased by 0.19% using Equation (3), where *β* is FWHM and *θ* is the Bragg angle [29,36].

(3) $$\varepsilon =\frac{\beta }{4tan\theta }$$

According to Bragg’s law, this low-angle shift corresponds to an increase in the d-spacing, which signifies the presence of tensile strain along the [001] direction. The weak van der Waals forces between the layers were easily altered, and the in-plane strain caused negligible variations in the sulfur-to-sulfur distance along the z-axis [37,38]. Figure 2e presents the band structure of few-layered MoS_2_, calculated using density functional theory (DFT), illustrating that the K-Γ indirect bandgap decreases from 1.15 eV in flat MoS_2_ to 1.14 eV in strained MoS_2_. This reduction of 10 meV results from the application of 0.8% uniaxial tensile strain in-plane. This reduction was attributed to the elongation of the bonds between the Mo and S atoms, which reduced the orbital overlap. Furthermore, the reciprocal space distortion caused the Κ and Γ points of the Brillouin zone to shift, which reduced the energy difference between the valence band maximum and conduction band minimum [39]. The increased curvature also resulted in a decrease in the effective mass, which enhanced the carrier mobility [40,41]. These changes in the band structure are expected to facilitate carrier and vacancy movement during the ReRAM operation, thereby reducing the energy required for conduction path formation and contributing to low-power operation.

**Figure 3:**
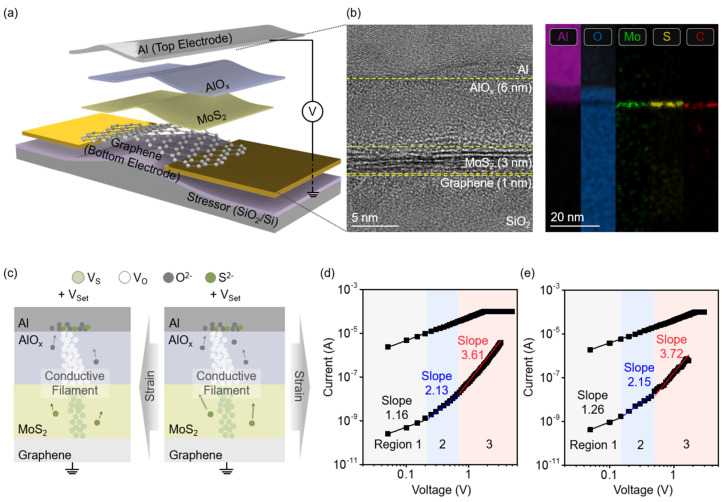
(**a**) Schematic illustration of a device based on strained MoS_2_ on a line-patterned SiO_2_/Si stressor. The bottom electrode is graphene, the resistive switching layer is strained MoS_2_ and AlO_x_, the top electrode is Al, and Au serves as the measurement electrode. (**b**) TEM and EDS images of strained device. (**c**) Comparison of the set mechanism between flat (left) and strained (right) devices. Double logarithmic I-V curve for the SCLC mechanism in HRS during set process: (**d**) flat, (**e**) strained devices.

To verify this hypothesis, ReRAM devices were fabricated using MoS_2_ under applied stress on a stressor and also using MoS_2_ on a flat substrate without internal stress for comparison. Figure 3a presents the schematics of the fabricated strain-induced-MoS_2_-based ReRAM devices; the fabrication process is detailed in the Experimental Section. The vertical structure of the device, confirmed through transmission electron microscopy (TEM) and energy-dispersive X-ray spectroscopy (EDS) (Figure 3b), comprised an Al top electrode, a 5 nm AlO_x_ layer, a 3 nm MoS_2_ resistive switching layer, and a graphene bottom electrode. Here, the AlO_x_ layer was formed using natural oxidation rather than deposition to retain the substrate effect of the strained MoS_2_ [42]. The oxide layer provided oxygen vacancies at the electrode/oxide interface, which enabled forming-free switching during the ReRAM operation [43,44]. Figure 3c illustrates the operating mechanism of the bipolar non-volatile ReRAM device based on a valence change mechanism (VCM). During the setting process, when a positive voltage was applied to the Al top electrode, negatively charged oxygen and sulfur ions migrated towards the top electrode, leaving behind vacancies. The vacancies in the AlO_x_ and MoS_2_ layers acted as charge trap sites and facilitated the formation of conductive filaments that provided electron transport pathways between the top and bottom electrodes, which resulted in a low-resistance state (LRS). Conversely, during the reset process, a negative voltage applied to the top electrode drew the ions back into the vacancies, which disrupted the conductive path and transitioned the device to a high-resistance state (HRS) (Figure S6a). Like the operation mechanism, the key factor in ReRAM is the ion movement driven by the applied voltage and the ease and reproducibility of the conductive filament formation. Notably, the strain-induced-MoS_2_-based ReRAM with an expanded atomic distance in the horizontal direction suggests a more efficient vertical migration of the charged ions under an external electric field. This can be demonstrated by a comparative analysis of the set process based on the flat (Figure 3d) and strained MoS_2_ devices (Figure 3e) using the space-charge-limited conduction (SCLC) mechanism [45,46,47]. In region 1 (V < V_tr_, transition voltage) of HRS, the device exhibited Ohmic behavior (I ∝ V), which was attributed to the dominance of the thermally generated carriers over the injected carries. As the applied electric field increased (V_tr_ < V < V_TFL_, trap-filled limit voltage), corresponding to region 2, the carriers were trapped at the vacancies, leading to a resistance reduction following Child’s law (I ∝ V^2^). Finally, in region 3 (V > V_TFL_), under a high electric field, the injected carriers saturated the available trap sites. Once all the vacancies were filled, a large number of free carriers could move through the resistive switching layer, causing a steep rise in the current, thereby transitioning the device to the LRS. The LRS exhibited Ohmic conductions, which indicates the formation of conductive filaments within the device. In the flat-MoS_2_-based device, the V_tr_ and V_TFL_ were 0.2 V and 0.64 V, respectively. In contrast, the strained-MoS_2_-based devices exhibited a V_tr_ of 0.15 V and V_TFL_ of 0.5 V. This demonstrates that internal tensile strain reduced the V_tr_ and V_TFL_ by approximately 25% and 22%, respectively. This reduction can be attributed to a decrease in the bandgap, which enabled electron injection and trap filling at lower voltages. Additionally, in region 3 (the TFL region), the linear fit of the I-V curve revealed that the slope of the strained device was 0.11 greater than that of the flat device. This can be attributed to the tensile strain, including lattice space relaxation, carrier effective mass reduction, and electron mobility enhancement [48,49].

**Figure 4:**
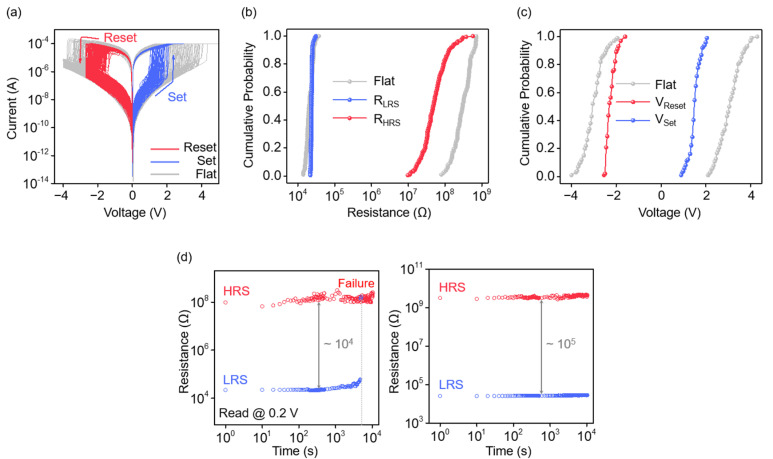
(**a**) I-V curves for the set and reset processes over 150 cycles for flat (gray) and strained devices (red and blue). (**b**) Cumulative probability distribution of the LRS and HRS for flat (gray) and strained (red and blue) devices. (**c**) Cumulative probability distribution of the V_Set_ and V_Reset_ for flat (gray) and strained (red and blue) devices. (**d**) Retention time measurement of flat (left) and strained (right) devices at a read voltage of 0.2 V.

Figure 4a shows the set and reset results over 150 cycles for the flat (gray) and strained devices (red and blue). Both devices demonstrated stable operation throughout the cycling process with a noticeable difference in the operating voltages. To demonstrate the cycle-to-cycle uniformity, the cumulative probabilities of the resistance in the LRS (R_LRS_), resistance in the HRS (R_HRS_) (Figure 4b), set voltage (V_Set_), and reset voltage (V_Reset_) (Figure 4c) were calculated based on 150 consecutive switching cycles. The average R_HRS_ and R_LRS_ of the flat device were measured at 3.41 × 10^8^ Ω and 2.13 × 10^4^ Ω, respectively, while the strained device exhibited an average R_HRS_ of 6.95 × 10^7^ Ω and an R_LRS_ of 2.36 × 10^4^ Ω. Both the devices exhibited a narrow cumulative probability distribution for resistance, which indicates a uniform alignment of vacancies along the conductive paths. Comparing the operating voltages, the flat device operated within the range of −4 to 4 V, with an average V_Set_ of 3.04 V and V_Reset_ of −3.03 V (Figure 4c, gray). In contrast, the strained device operated within a narrower range of −2.5 to 2.5 V, with an average V_Set_ of 1.48 V and V_Reset_ of −2.27 V (Figure 4c, red and blue). Statistical data from measurements of 100 flat devices and 100 strained devices show that the flat devices operate within a ±3.7 V range, while the strained devices operate within a ±2 V range (Figure S7). This consistency demonstrates the reproducibility and reliability of our experimental results. The reduced range of the strained device can be attributed to the structural deformation of the crystal, in which the weakened bonding lowers the energy required for vacancy diffusion and enables the formation of conductive paths at lower voltages [50,51]. Furthermore, the narrower distribution suggests minimal voltage fluctuation during the filament rupture and reformation processes, which is advantageous for consistent and reliable device responses. Figure 4d shows the retention characteristics monitored at a read voltage of 0.2 V to evaluate the resistance maintenance ability over time. For the flat device (Figure 4d, left), the return to the HRS (failure) at 5.2 × 10^3^ s can be attributed to the stabilization process, where ions recombine with vacancies, leading to filament rupture. In contrast, the strained device (Figure 4d, right) maintained a stable resistance state for up to 10^4^ s, showing a two-fold improvement compared with the flat device, which demonstrates its potential for application in non-volatile memory. Table S1 provides a comparative analysis of this study with other recently reported MoS_2_-based devices. These results highlight the advantages of introducing tensile strain and lowering the operating voltage while generating uniform improvements across various performances in comparison to existing works.

## 4. Conclusions

A novel strain engineering method was developed to induce permanent tensile strain in MoS_2_ using a stressor substrate with a controlled surface morphology. Analyses revealed that the MoS_2_, conformally in contact with the periodic topography of the stressor, contained a tensile strain of approximately 0.8%. This induced strain in MoS_2_, due to Mo-S bond elongation, affects its physical properties in two main ways. Firstly, strain reduces the bandgap by approximately 10 meV, which lowers the energy needed for charge carriers to engage in conduction. Secondly, strain reduces orbital overlap which decreases the effective mass and increases the mobility of charge carriers. This improvement enables more efficient movement of ions and vacancies, allowing conduction path formation and rupture at lower voltages. This approach enhances the functional versatility of 2D materials without any additional doping. By integrating this strain-engineered MoS_2_ into ReRAM, the fundamental unit of neuromorphic devices, a significant reduction in the operating voltage was demonstrated, which was lowered from ±3 V to ±2 V level compared to the non-strained-MoS_2_-based device. Furthermore, the strain-induced MoS_2_ device achieved a 22% reduction in the trap-filled limit voltage (V_TFL_). Owing to the more stable formation and rupture of the conductive filaments in the strain-engineered MoS_2_ layer, the device exhibited improved uniformity in resistance distribution and extended retention times, which indicates enhanced stability for memory applications. These findings highlight the potential of strain-engineered MoS_2_ in the development of energy-efficient, non-volatile memory devices, opening new opportunities for 2D materials in next-generation semiconductor technologies and neuromorphic computing.

## Data Availability

Data are contained within the article and Supplementary Materials.

## Supplementary Materials

The following supporting information can be downloaded at: https://www.mdpi.com/article/10.3390/nano14231872/s1, Figure S1: Fabrication conditions and surface morphology of stressors; Figure S2: Strain distribution of stressor; Figure S3: RMS roughness of original and stressor substrate; Figure S4: Thickness of graphene and changes in RMS roughness after transfer process; Figure S5: Raman spectra; Figure S6: Operating mechanism of ReRAM and representation of multi-bit capability; Figure S7: Statistical analysis of 100 flat and 100 strained devices; Table S1: Comparison of device performance of this study with other recently reported MoS_2_-based Devices. Refs. [52,53,54,55,56,57,58] are cited in the supplementary materials.

## References

[1] Welser J., Hoyt J., Takagi S.-I., Gibbons J. Strain dependence of the performance enhancement in strained-Si n-MOSFETs. Proceedings of the 1994 IEEE International Electron Devices Meeting.

[2] Rim K., Welser J., Hoyt J., Gibbons J. Enhanced hole mobilities in surface-channel strained-Si p-MOSFETs. Proceedings of the International Electron Devices Meeting.

[3] Ghani T., Armstrong M., Auth C., Bost M., Charvat P., Glass G., Hoffmann T., Johnson K., Kenyon C., Klaus J. A 90nm high volume manufacturing logic technology featuring novel 45nm gate length strained silicon CMOS transistors. Proceedings of the IEEE International Electron Devices Meeting 2003.

[4] Lemme M.C., Wagner S., Lee K., Fan X., Verbiest G.J., Wittmann S., Lukas S., Dolleman R.J., Niklaus F., van der Zant H.S. (2020). Nanoelectromechanical sensors based on suspended 2D materials. Research.

[5] Conley H.J., Wang B., Ziegler J.I., Haglund Jr R.F., Pantelides S.T., Bolotin K.I. (2013). Bandgap engineering of strained monolayer and bilayer MoS_2_. Nano Lett..

[6] He K., Poole C., Mak K.F., Shan J. (2013). Experimental demonstration of continuous electronic structure tuning via strain in atomically thin MoS_2_. Nano Lett..

[7] Gu Z., Zhang T., Luo J., Wang Y., Liu H., Chen L., Liu X., Yu W., Zhu H., Sun Q.-Q. (2020). MoS_2_-on-AlN enables high-performance MoS_2_ field-effect transistors through strain engineering. ACS Appl. Mater. Interfaces.

[8] Li Z., Lv Y., Ren L., Li J., Kong L., Zeng Y., Tao Q., Wu R., Ma H., Zhao B. (2020). Efficient strain modulation of 2D materials via polymer encapsulation. Nat. Commun..

[9] Datye I.M., Daus A., Grady R.W., Brenner K., Vaziri S., Pop E. (2022). Strain-enhanced mobility of monolayer MoS_2_. Nano Lett..

[10] Pang F., Cao F., Lei L., Meng L., Ye S., Xing S., Guo J., Dong H., Hussain S., Gu S. (2021). Strain-engineered rippling and manipulation of single-layer WS2 by atomic force microscopy. J. Phys. Chem. C.

[11] Ahn G.H., Amani M., Rasool H., Lien D.-H., Mastandrea J.P., Ager III J.W., Dubey M., Chrzan D.C., Minor A.M., Javey A. (2017). Strain-engineered growth of two-dimensional materials. Nat. Commun..

[12] Wang J., Han M., Wang Q., Ji Y., Zhang X., Shi R., Wu Z., Zhang L., Amini A., Guo L. (2021). Strained epitaxy of monolayer transition metal dichalcogenides for wrinkle arrays. ACS Nano.

[13] Martella C., Mennucci C., Cinquanta E., Lamperti A., Cappelluti E., Buatier de Mongeot F., Molle A. (2017). Anisotropic MoS_2_ nanosheets grown on self-organized nanopatterned substrates. Adv. Mater..

[14] Xie S., Tu L., Han Y., Huang L., Kang K., Lao K.U., Poddar P., Park C., Muller D.A., DiStasio R.A. (2018). Coherent, atomically thin transition-metal dichalcogenide superlattices with engineered strain. Science.

[15] Castellanos-Gomez A., Roldán R., Cappelluti E., Buscema M., Guinea F., Van Der Zant H.S., Steele G.A. (2013). Local strain engineering in atomically thin MoS_2_. Nano Lett..

[16] Li H., Contryman A.W., Qian X., Ardakani S.M., Gong Y., Wang X., Weisse J.M., Lee C.H., Zhao J., Ajayan P.M. (2015). Optoelectronic crystal of artificial atoms in strain-textured molybdenum disulphide. Nat. Commun..

[17] Bae S., Kim H., Lee Y., Xu X., Park J.-S., Zheng Y., Balakrishnan J., Lei T., Ri Kim H., Song Y.I. (2010). Roll-to-roll production of 30-inch graphene films for transparent electrodes. Nat. Nanotechnol..

[18] Jain A., Bharadwaj P., Heeg S., Parzefall M., Taniguchi T., Watanabe K., Novotny L. (2018). Minimizing residues and strain in 2D materials transferred from PDMS. Nanotechnology.

[19] Wang X., Fan W., Fan Z., Dai W., Zhu K., Hong S., Sun Y., Wu J., Liu K. (2018). Substrate modified thermal stability of mono-and few-layer MoS_2_. Nanoscale.

[20] Kim H.G., Kihm K.D., Lee W., Lim G., Cheon S., Lee W., Pyun K.R., Ko S.H., Shin S. (2017). Effect of graphene-substrate conformity on the in-plane thermal conductivity of supported graphene. Carbon.

[21] Fitch J., Bjorkman C., Lucovsky G., Pollak F., Yin X. (1989). Intrinsic stress and stress gradients at the SiO_2_/Si interface in structures prepared by thermal oxidation of Si and subjected to rapid thermal annealing. J. Vac. Sci. Technol. B Microelectron. Process. Phenom..

[22] EerNisse E. (1979). Stress in thermal SiO_2_ during growth. Appl. Phys. Lett..

[23] Park S., Song J., Kim T.K., Choi K.H., Hyeong S.K., Ahn M., Kim H.R., Bae S., Lee S.K., Hong B.H. (2022). Photothermally Crumpled MoS_2_ Film as an Omnidirectionally Stretchable Platform. Small Methods.

[24] Serrano J.R., Cahill D.G. (2002). Micron-scale buckling of SiO_2_ on Si. J. Appl. Phys..

[25] Hobart K., Kub F., Fatemi M., Twigg M., Thompson P., Kuan T., Inoki C. (2000). Compliant substrates: A comparative study of the relaxation mechanisms of strained films bonded to high and low viscosity oxides. J. Electron. Mater..

[26] Hu Y., Zhang F., Titze M., Deng B., Li H., Cheng G.J. (2018). Straining effects in MoS_2_ monolayer on nanostructured substrates: Temperature-dependent photoluminescence and exciton dynamics. Nanoscale.

[27] Li H., Zhang Q., Yap C.C.R., Tay B.K., Edwin T.H.T., Olivier A., Baillargeat D. (2012). From bulk to monolayer MoS_2_: Evolution of Raman scattering. Adv. Funct. Mater..

[28] Lee J.-U., Woo S., Park J., Park H.C., Son Y.-W., Cheong H. (2017). Strain-shear coupling in bilayer MoS_2_. Nat. Commun..

[29] Kolhe P., Thorat A., Phatangare A., Jadhav P., Dalvi S., Dhole S., Dahiwale S. (2022). Strain induced study on MoS_2_ thin films due to ion and gamma irradiation. J. Alloys Compd..

[30] Gan Y., Zhao H. (2016). Chirality and vacancy effect on phonon dispersion of MoS_2_ with strain. Phys. Lett. A.

[31] Jiang J.-W. (2014). Phonon bandgap engineering of strained monolayer MoS_2_. Nanoscale.

[32] Tornatzky H., Gillen R., Uchiyama H., Maultzsch J. (2019). Phonon dispersion in MoS_2_. Phys. Rev. B.

[33] Chang C.-H., Fan X., Lin S.-H., Kuo J.-L. (2013). Orbital analysis of electronic structure and phonon dispersion in MoS_2_, MoSe_2_, WS_2_, and WSe2 monolayers under strain. Phys. Rev. B-Condens. Matter Mater. Phys..

[34] Mignuzzi S., Pollard A.J., Bonini N., Brennan B., Gilmore I.S., Pimenta M.A., Richards D., Roy D. (2015). Effect of disorder on Raman scattering of single-layer MoS_2_. Phys. Rev. B.

[35] Vandalon V., Sharma A., Perrotta A., Schrode B., Verheijen M.A., Bol A.A. (2019). Polarized Raman spectroscopy to elucidate the texture of synthesized MoS_2_. Nanoscale.

[36] Yang L., Cui X., Zhang J., Wang K., Shen M., Zeng S., Dayeh S.A., Feng L., Xiang B. (2014). Lattice strain effects on the optical properties of MoS_2_ nanosheets. Sci. Rep..

[37] Miao Y.-P., Ma F., Huang Y.-H., Xu K.-W. (2015). Strain effects on electronic states and lattice vibration of monolayer MoS_2_. Phys. E Low-Dimens. Syst. Nanostruct..

[38] Li T. (2012). Ideal strength and phonon instability in single-layer MoS_2_. Phys. Rev. B-Condens. Matter Mater. Phys..

[39] Bendavid L.I., Zhong Y., Che Z., Konuk Y. (2022). Strain-engineering in two-dimensional transition metal dichalcogenide alloys. J. Appl. Phys..

[40] Peelaers H., Van de Walle C.G. (2012). Effects of strain on band structure and effective masses in MoS_2_. Phys. Rev. B-Condens. Matter Mater. Phys..

[41] Phuc H.V., Hieu N.N., Hoi B.D., Hieu N.V., Thu T.V., Hung N.M., Ilyasov V.V., Poklonski N.A., Nguyen C.V. (2018). Tuning the Electronic Properties, Effective mass and carrier mobility of MoS_2_ monolayer by strain engineering: First-principle calculations. J. Electron. Mater..

[42] Nguyen L., Hashimoto T., Zakharov D.N., Stach E.A., Rooney A.P., Berkels B., Thompson G.E., Haigh S.J., Burnett T.L. (2018). Atomic-scale insights into the oxidation of aluminum. ACS Appl. Mater. Interfaces.

[43] Kindsmüller A., Meledin A., Mayer J., Waser R., Wouters D.J. (2019). On the role of the metal oxide/reactive electrode interface during the forming procedure of valence change ReRAM devices. Nanoscale.

[44] Kumar R., Kalaboukhov A., Weng Y.-C., Rathod K., Johansson T., Lindblad A., Kamalakar M.V., Sarkar T. (2024). Vacancy-Engineered Nickel Ferrite Forming-Free Low-Voltage Resistive Switches for Neuromorphic Circuits. ACS Appl. Mater. Interfaces.

[45] Yen T.J., Chin A., Gritsenko V. (2020). High performance all nonmetal SiNx resistive random access memory with strong process dependence. Sci. Rep..

[46] Kumari C., Varun I., Tiwari S.P., Dixit A. (2018). Robust non-volatile bipolar resistive switching in sol-gel derived BiFeO_3_ thin films. Superlattices Microstruct..

[47] Kwan C.-P., Street M., Mahmood A., Echtenkamp W., Randle M., He K., Nathawat J., Arabchigavkani N., Barut B., Yin S. (2019). Space-charge limited conduction in epitaxial chromia films grown on elemental and oxide-based metallic substrates. AIP Adv..

[48] Hosseini M., Elahi M., Pourfath M., Esseni D. (2015). Strain induced mobility modulation in single-layer MoS_2_. J. Phys. D Appl. Phys..

[49] Khan M., Tripathi M.N., Tripathi A. (2022). Strain-induced structural, elastic, and electronic properties of 1L-MoS_2_. J. Mater. Res..

[50] Al-Hamadany R., Goss J., Briddon P., Mojarad S.A., O’Neill A., Rayson M. (2013). Impact of tensile strain on the oxygen vacancy migration in SrTiO_3_: Density functional theory calculations. J. Appl. Phys..

[51] Kim K., Siegel D.J. (2019). Correlating lattice distortions, ion migration barriers, and stability in solid electrolytes. J. Mater. Chem. A.

[52] Yan H., Zhuang P., Li B., Ye T., Zhou C., Chen Y., Li T., Cai W., Yu D., Liu J. (2024). Metal Penetration and Grain Boundary in MoS_2_ Memristors. Adv. Electron. Mater..

[53] Choudhary S., Soni M., Sharma S.K. (2019). Low voltage & controlled switching of MoS_2_-GO resistive layers based ReRAM for non-volatile memory applications. Semicond. Sci. Technol..

[54] Lei X., Zhu X., Wang H., Zhang H., Zhai C., Wang S., Yan J., Zhao W. (2023). Nonvolatile and volatile resistive switching characteristics in MoS_2_ thin film for RRAM application. J. Alloys Compd..

[55] Krishnaprasad A., Dev D., Shawkat M.S., Martinez-Martinez R., Islam M.M., Chung H.-S., Bae T.-S., Jung Y., Roy T. (2023). Graphene/MoS_2_/SiO_x_ memristive synapses for linear weight update. Npj 2d Mater. Appl..

[56] Kim M., Ge R., Wu X., Lan X., Tice J., Lee J.C., Akinwande D. (2018). Zero-static power radio-frequency switches based on MoS_2_ atomristors. Nat. Commun..

[57] Belete M., Kataria S., Turfanda A., Vaziri S., Wahlbrink T., Engström O., Lemme M.C. (2020). Nonvolatile resistive switching in nanocrystalline molybdenum disulfide with ion-based plasticity. Adv. Electron. Mater..

[58] Bhattacharjee S., Caruso E., McEvoy N., Ó Coileáin C., O’Neill K., Ansari L., Duesberg G.S., Nagle R., Cherkaoui K., Gity F. (2020). Insights into multilevel resistive switching in monolayer MoS_2_. ACS Appl. Mater. Interfaces.

