# Supplementary material for: Permanent Strain Engineering of Molybdenum Disulfide Using Laser-Driven Stressors for Energy-Efficient Resistive Switching Memory Devices
Source: Nanomaterials (Basel). 2024 Nov 22;14(23):1872. doi: 10.3390/nano14231872 (PMC11643195; doi:10.3390/nano14231872)
Supplement: Supplementary file 1 [file nanomaterials-14-01872-s001.zip › nanomaterials-3293524-supplementary.pdf]

Supporting Information

Permanent Strain Engineering of Molybdenum Disulfide Using Laser-Driven Stressors for Energy-Efficient Resistive Switching Memory Devices

Heeyoon Jang[1], Seok-Ki Hyeong[2], Byeongjin Park[3], Tae Wook Kim[4,5], Sukang Bae[2,5], Sung Kyu Jang[6], Yonghun Kim[3*] and Seoung-Ki Lee[1*]*

[1]School of Material Science and Engineering, Pusan National University, Busan 46241, Republic of Korea

[2]Functional Composite Materials Research Center, Institute of Advanced Composite Materials, Korea Institute of Science and Technology (KIST), 92 Chudong-ro, Bongdong-eup, Wanju-gun 55324, Republic of Korea

[3]Energy and Environment Materials Research Division, Korea Institute of Materials Science (KIMS), 797 Changwondaero, Sungsan-gu, Changwon 51508, Republic of Korea

[4]Department of Flexible and Printable Electronics, Jeonbuk National University, Jeonju-si 54896, Republic of Korea

[5]Department of JBNU-KIST Industry-Academia Convergence Research, Jeonbuk National University, Jeonju-si 54896, Republic of Korea

[6]Electronic Convergence Material and Device Research Center, Korea Electronics Technology Institute, Seongnam 13509, Republic of Korea

*Corresponding Author

E-mail: skjang@keti.re.kr (S.K.J.); kyhun09@kims.re.kr (Y.K.); ifriend@pusan.ac.kr (S.-K.L.)

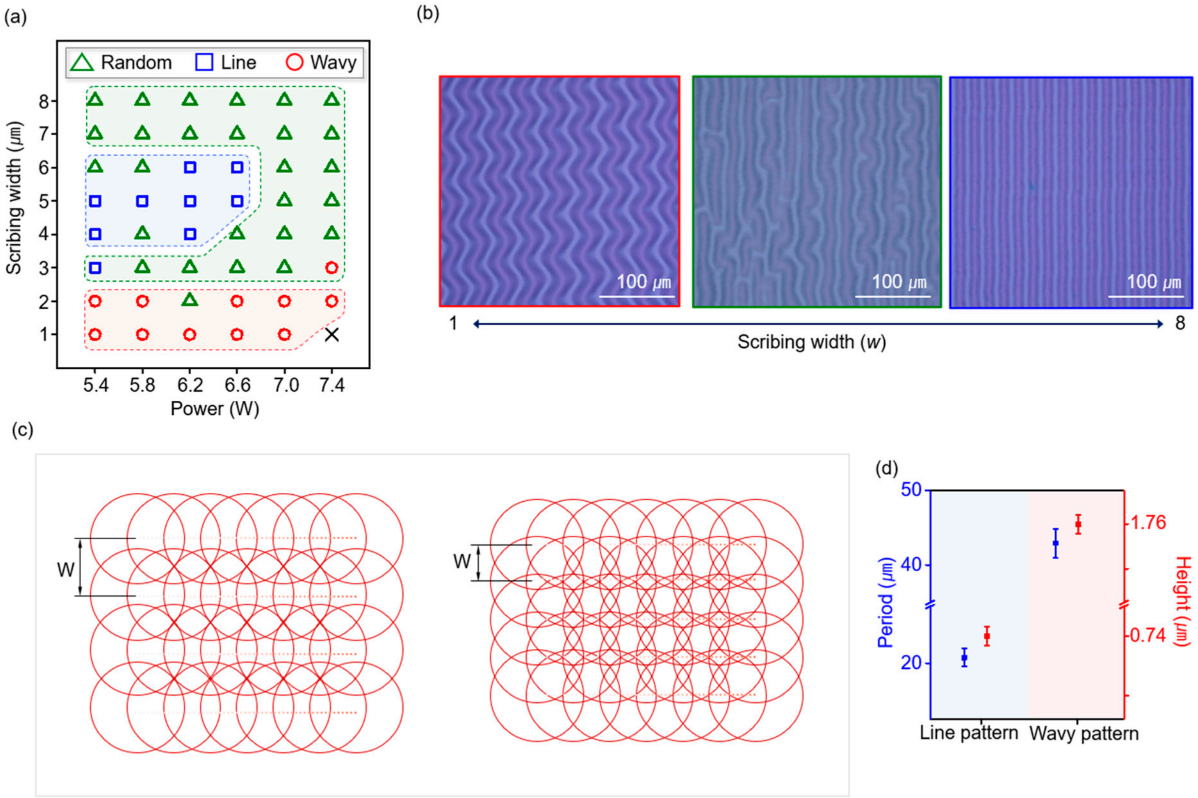

Figure S1. a) Representation of SiO_2/Si stressor patterns formed under different laser power and line width conditions. b) Progression of pattern morphology from line (blue) to random (green) to wavy (red) as the scribing width decreases. c) Adjustment of laser spot overlap by controlling pulsed laser scribing width (*w*). The wavy pattern (right) demonstrates a smaller w compared to the line pattern (left). d) Period and height of line and wavy patterns.

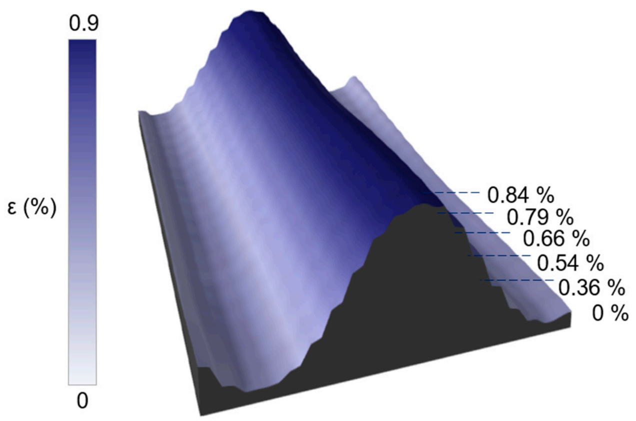

Figure S2. The strain distribution within the line pattern stressor ranges from 0.84% to 0%. Strain effects are analyzed based on measurements at the peak of hill structures, where the strain is most pronounced.

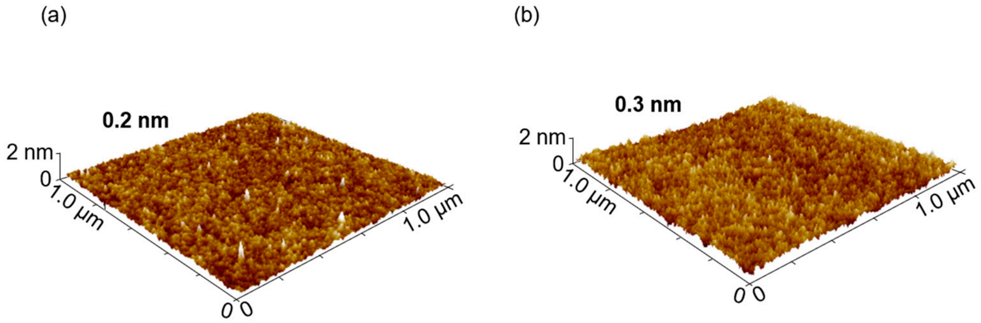

Figure S3. a) RMS surface roughness measurement of the cleaned, flat SiO_2/Si wafer prior to laser irradiation. b) RMS surface roughness measurement of the SiO_2/Si stressor with hill structures formed after laser irradiation, indicating that laser irradiation does not impact surface roughness.

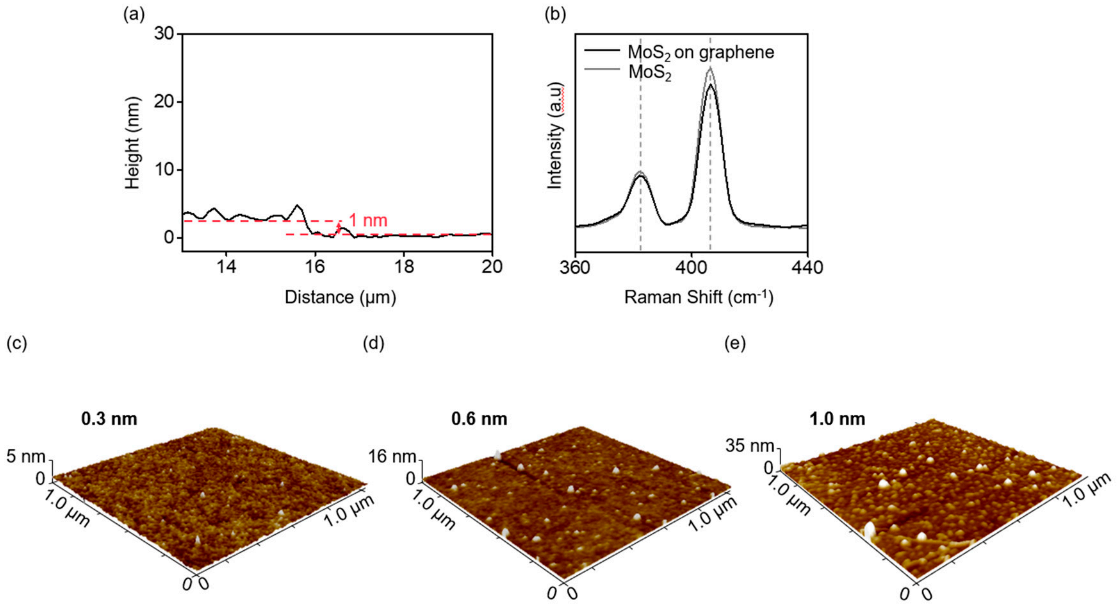

Figure S4. a) Measurement of graphene thickness as synthesized on the substrate. b) Comparison of MoS_2 Raman Spectra on stressor with (black) and without (gray) graphene. c) Analysis of surface roughness for the original substrate, graphene on substrate, and MoS_2 on graphene, providing insight into layer conformity and interface quality post-transfer.

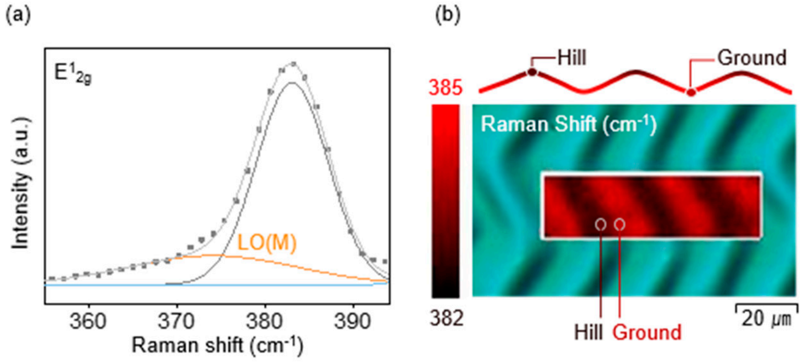

Figure S5. a) Magnified view of the E^1_{2g} Raman peak of flat MoS_2, showing no distinct splitting between the LO and TO modes. b) Raman mapping image (50 × 20 μm^2) of strained-MoS_2 on wavy pattern stressor.

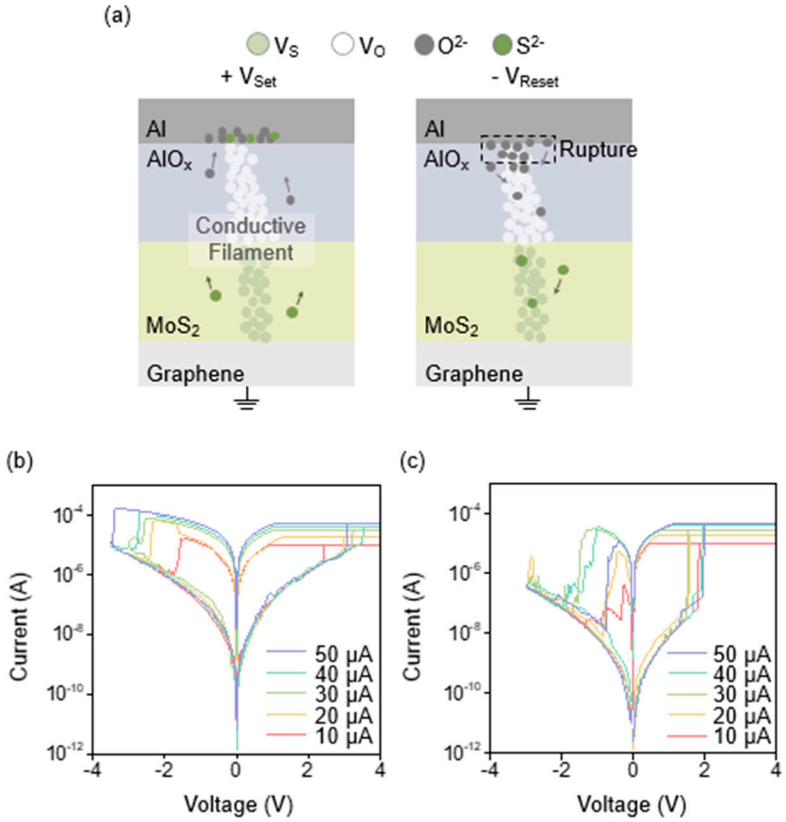

Figure S6. a) Schematic illustration of the set (left) and reset (right) mechanism based VCM, showing oxygen and sulfur vacancies forming and breaking conductive filaments. Multi-bit operation of b) flat and c) strained devices by adjusting current compliance.

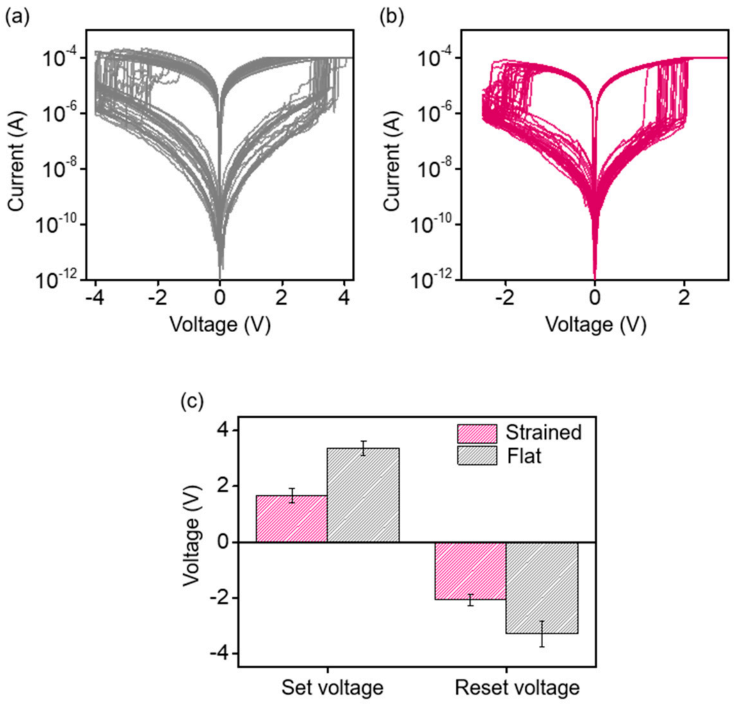

Figure S7. I-V characteristics of 100 individual a) flat and b) strained ReRAM devices, demonstrating device-to-device consistency within this configuration. c) Statistical analysis of set and reset voltage extracted from the 100 flat and 100 strained devices, illustrating that the strained devices exhibit significantly lower operating voltages compared to the flat devices.

Structure	Endurance [cycle #]	Retention [s]	Switching ratio	HRS [Ω]	LRS [Ω]	Set [V]	Reset [V]	Reference
Au/MoS_2/Au	100	10^5	10^2	$5x10^3$	10	1	-1	[1]
Al/MoS_2-GO/Al	25	10^4	10^2	10^6	10^4	-0.35	0.5	[2]
Cu/MoS_2/ITO	10^3	10^4	10	10^4	10^3	1.5	-1.5	[3]
Graphene/MoS_2/SiO_x/Ni	50	10^3	10^4	10^9	10^5	4.5	-3	[4]
Au/MoS_2/Au	20	10^4	10^2	10^3	10	1.2	-1	[5]
Si/MoS_2/Cr/Au	140	$2.5x10^3$	10	$2.4x10^3$	$1.2x10^3$	-3.5	4	[6]
Au/MoS_2/Au	20	10^4	10^6	10^9	10^3	3	-2	[7]
Graphene/tensile strained MoS_2/AlO_x/Al	150	10^4	10^4	10^8	10^4	1.5	-2	This work

Table S1. Comparison of Device Performance of This Study with Other Recently Reported MoS_2 -Based Devices

References:

1. H. Yan; P. Zhuang; B. Li; T. Ye; C. Zhou; Y. Chen; T. Li; W. Cai; D. Yu; J. Liu. Metal Penetration and Grain Boundary in MoS_2 Memristors, *Advanced Electronic Materials*, 2400264.
2. S. Choudhary; M. Soni; S. K. Sharma. Low voltage & controlled switching of MoS_2-GO resistive layers based ReRAM for non-volatile memory applications, *Semiconductor Science and Technology,* **2019**, *34*, *8*, 085009.
3. X. Lei; X. Zhu; H. Wang; Y. Dai; H. Zhang; C. Zhai; S. Wang; J. Yan; W. Zhao. Nonvolatile and volatile resistive switching characteristics in MoS_2 thin film for RRAM application, *Journal of Alloys and Compounds,* **2023**, *969*, 172443.
4. A. Krishnaprasad; D. Dev; M. S. Shawkat; R. Martinez-Martinez; M. M. Islam; H.-S. Chung; T.-S. Bae; Y. Jung; T. Roy. Graphene/MoS_2/SiO_x memristive synapses for linear weight update, *npj 2D Materials and Applications,* **2023**, *7*, *1*, 22.
5. M. Kim; R. Ge; X. Wu; X. Lan; J. Tice; J. C. Lee; D. Akinwande. Zero-static power radio-frequency switches based on MoS_2 atomristors, *Nature communications,* **2018**, *9*, *1*, 2524.
6. M. Belete; S. Kataria; A. Turfanda; S. Vaziri; T. Wahlbrink; O. Engström; M. C. Lemme. Nonvolatile resistive switching in nanocrystalline molybdenum disulfide with ion-based plasticity, *Advanced Electronic Materials,* **2020**, *6*, *3*, 1900892.
7. S. Bhattacharjee; E. Caruso; N. McEvoy; C. Ó Coileáin; K. O'Neill; L. Ansari; G. S. Duesberg; R. Nagle; K. Cherkaoui; F. Gity. Insights into multilevel resistive switching in monolayer MoS_2, *ACS applied materials & interfaces,* **2020**, *12*, *5*, 6022-6029.
